# Tracking Time Evolution of Collective Attention Clusters in Twitter: Time Evolving Nonnegative Matrix Factorisation

**DOI:** 10.1371/journal.pone.0139085

**Published:** 2015-09-29

**Authors:** Shota Saito, Yoshito Hirata, Kazutoshi Sasahara, Hideyuki Suzuki

**Affiliations:** 1 Department of Mathematical Informatics, Graduate School of Information Science and Technology, The University of Tokyo, Tokyo, Japan; 2 Institute of Industrial Science, The University of Tokyo, Tokyo, Japan; 3 Department of Complex Systems Science, Graduate School of Information Science, Nagoya University, Nagoya, Japan; University of Rijeka, CROATIA

## Abstract

Micro-blogging services, such as Twitter, offer opportunities to analyse user behaviour. Discovering and distinguishing behavioural patterns in micro-blogging services is valuable. However, it is difficult and challenging to distinguish users, and to track the temporal development of collective attention within distinct user groups in Twitter. In this paper, we formulate this problem as tracking matrices decomposed by Nonnegative Matrix Factorisation for time-sequential matrix data, and propose a novel extension of Nonnegative Matrix Factorisation, which we refer to as *Time Evolving Nonnegative Matrix Factorisation* (*TENMF*). In our method, we describe users and words posted in some time interval by a matrix, and use several matrices as time-sequential data. Subsequently, we apply Time Evolving Nonnegative Matrix Factorisation to these time-sequential matrices. TENMF can decompose time-sequential matrices, and can track the connection among decomposed matrices, whereas previous NMF decomposes a matrix into two lower dimension matrices arbitrarily, which might lose the time-sequential connection. Our proposed method has an adequately good performance on artificial data. Moreover, we present several results and insights from experiments using real data from Twitter.

## Introduction

Social behaviour of human beings has been an important research topic, and as the Internet grows, we can observe the behaviour also through the Internet data [1]. Particularly the use of social networking sites in daily lives are increasing [2]. Therefore these websites have a large amount of traces of human behaviour, and using these traces many interesting phenomena have been revealed [3–8]. Remarkably, recent growing micro-blogging services such as Twitter highly reflect human social behaviour, due to their real-time nature. Twitter is an extremely popular social networking site, consisting of over 250 million users, where the users can post a message about any topic within 140 characters, called a tweet. Collective attention is one of the valuable phenomena inherent to Twitter [9, 10]. Collective attention describes a situation in which a certain amount of people talk about a certain trending topic [11].

If we can catch what is an emerging collective attention, who is talking about it, and how it evolves or shrinks, then this attempt would be a precious commodity for social studies to analyse time-evolution and transition of human collective behaviour on web, which can be applied even to marketing businesses. For example, if a word such as a sports drink name becomes a trending topic in Twitter —not only among people who like to participate in sports, but also among people who like alcoholic drinks for the reason that it would be helpful to overcome hangovers by replacing water efficiently—, then businesspeople in charge of the sports drink marketing might be able to plan a promotion campaign aimed to not only sports shops but also liquor shops. This paper would aid such kinds of marketing activities.

Concerning the analysis of collective attention, the first question is how we classify users talking about topics of collective attention. For this question we propose to apply the Nonnegative Matrix Factorisation (NMF) proposed by Lee and Seung [12], one of the matrix decomposition methods. NMF can decompose the whole collection of data represented by a matrix into several parts. We propose to apply NMF to a matrix that is the counts of how often words were posted by users in Twitter. By conducting this procedure, we can group the users into several semantic groups, by words that characterise the content posted by the users. By analysing the grouped words, we can interpret what kind of user group mentions the topic of collective attention.

The next question is how we track the time evolution of collective attention. We propose *Time Evolving Nonnegative Matrix Factorisation (TENMF)* to track the time evolution of topics within clusters. TENMF can decompose time-sequential matrices, and can track the connection among decomposed matrices, whereas previous NMF decomposes a matrix into two lower dimension matrices arbitrarily, which might lose the time-sequential connection.

Our numerical experiments show that our approach is significantly better than the simple NMF method, from the perspective of tracking time sequential matrices. Moreover, the proposed method tracks more complex time-evolving of matrices. Furthermore, from 14 million tweets of 440 thousand users of Twitter, our method retrieves trends of some specific words in the clusters generated by words relating to the huge earthquake that occurred in 11th March 2011, and the release announcement of iPhone 4 held in 7th June 2010.

Our contribution in this paper is summarised as follows:

1. We focus on collective attention among distinguished user groups in social networking sites, while previous studies have focused on a whole system of social networking sites.
2. We propose a novel extension of Nonnegative Matrix Factorisation, Time Evolving Nonnegative Matrix Factorisation, for tracking temporal development of each cluster.
3. We apply TENMF for 14 million tweets from Twitter, and show that the trends of targeted words can be tracked in the reasonably corresponding cluster.

## Results

### Time Evolving Nonnegative Matrix Factorisation

Nonnegative Matrix Factorisation (NMF) is a matrix decomposition method, and has an advantage that it has an affinity with the intuition that we form a whole by adding its parts. Lee and Seung [12] reported NMF can decompose the whole of the data for a face into data for parts of a face such as eyes and nose. Specifically, NMF [12–15] decomposes a nonnegative matrix *V* into two lower dimensional nonnegative matrices *W* and *H* as follows:

V≈WH. (1)

The matrix *V* is an *m* × *n* matrix that represents a set of *m* dimensional data, such as images, graphs, and sounds. The matrix *W* is an *m* × *r* nonnegative matrix and contains as its columns the basis (features or clusters) of *V*. Each column of the *r* × *n* matrix *H* contains the component that gives the contribution of each basis, and encodes *W* to approximate *V*. Rank *r* is much smaller than *n* to reduce the dimension of *V*. The NMF algorithm to find *W* and *H*, proposed by Ref. [16], is described in the method section.

For Twitter data, if we assign words as rows and users as columns to a matrix which is counting how often the words were posted by the users in Twitter, then we can classify the words into several semantic groups using NMF. A similar technique using NMF has been demonstrated in the document clustering area: classifying words into semantic groups using a number of documents [12]. By analysing grouped words, we can interpret what kind of user group mentions topics of collective attention.

To describe the behaviour of twitter users, we define time sequential matrices. Let *t*_1_,*t*_2_,… be a sequence of times. We prepare a set of words 𝒲, and set of users 𝒰. The number of occurrences of the *i*th word in the *j*th user’s tweets between time *t*_*k*−1_ and *t*_*k*_ is represented by $v_{ij}^{\left(t_{k}\right)}$. Each column of *V* ^(*t*_*k*_)^ contains word counts for a particular user during a certain time, while each row of *V* ^(*t*_*k*_)^ represents counts of a particular word for users during a certain time.

**Fig 1:**
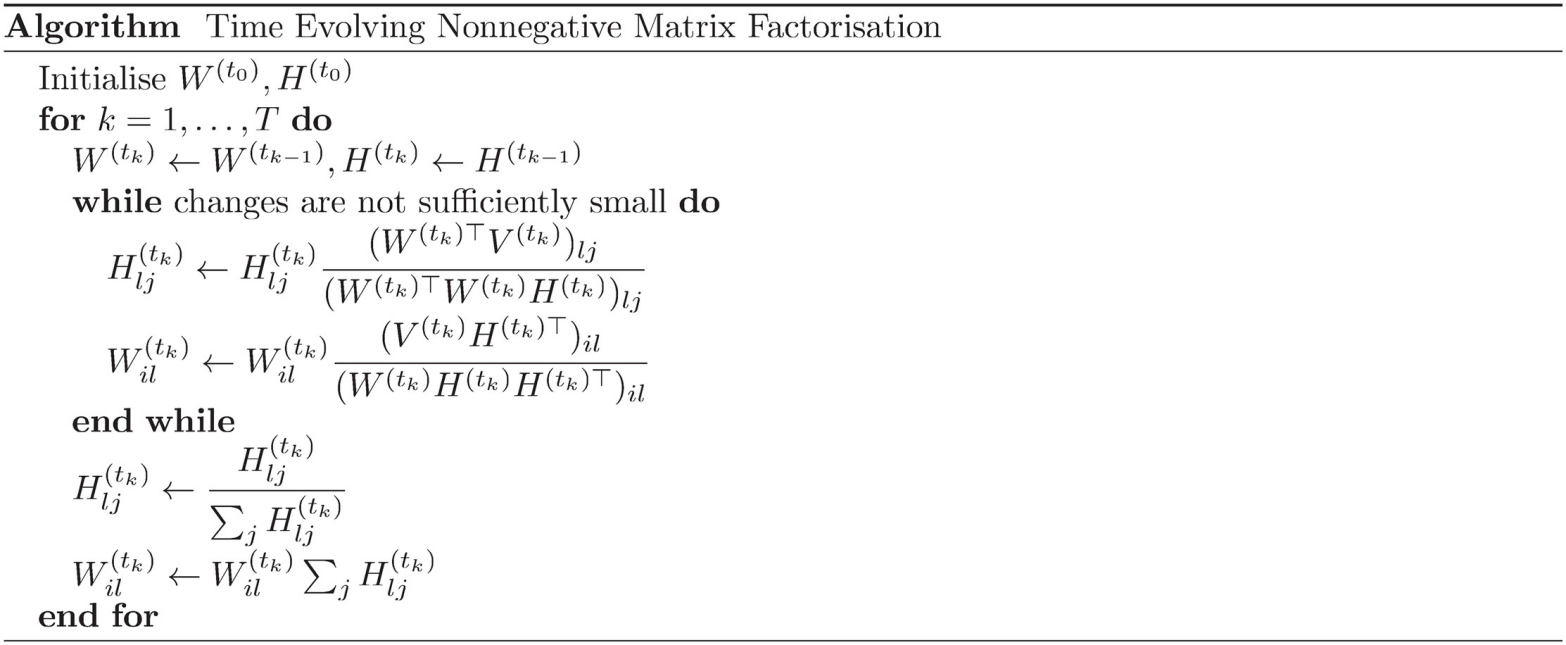
Iterative algorithm for Time Evolving Nonnegative Matrix Factorisation (TENMF). TENMF is an extension of Nonnegative Matrix Factorisation, to track the time-evolution of the *W* ^(*t*_*k*_)^s. Starting from initialised *W* ^(*t*_0_)^ and *H* ^(*t*_0_)^, we update as introduced in Ref. [16] and the method section. From the second time step, we use the decomposed result of one step back as initial conditions. Here we assume that two consecutive time-sequential matrices have a similarity. Since NMF converges to local optima, the process would result in a convergence to ‘near’ local optima, and would not lose temporal development information, i.e., preserve the similarity to the result of one step back.

If we simply apply NMF for time-sequential matrices, NMF loses pieces of information on the temporal development, because NMF decomposes the matrices arbitrarily. To solve this time-sequential problem, we propose here a Nonnegative Matrix Factorisation algorithm for time-evolving data. The idea behind *Time Evolving Nonnegative Matrix Factorisation (TENMF)* is to use *W* and *H* at time *t*_*k*_ to estimate *W* and *H* at time *t*_*k*+1_. Let us denote *W* and *H*, at time *t*_*k*_ by *W* ^(*t*_*k*_)^ and *H* ^(*t*_*k*_)^. NMF often converges to a local optimal solution, and the solution is highly affected by the initial condition [17–19]. Hence, if we set a seed as *W* ^(*t*_*k*_)^ and *H* ^(*t*_*k*_)^, the next (*W* ^(*t*_*k*+1_)^,*H* ^(*t*_*k*+1_)^) would converge to a ‘near’ local optimal solution, i.e. the locally optimal solution whose basin contains the current matrices. This convergence preserves the connection between (*W* ^(*t*_*k*_)^,*H* ^(*t*_*k*_)^) and (*W* ^(*t*_*k*+1_)^,*H* ^(*t*_*k*+1_)^). Applying the algorithm of NMF, introduced in the method section, the discussion above yields an algorithm as Fig 1.

A temporal development of the *l*th basis can be observed as

$$(w_{.l}^{\left(t_{1}\right)},w_{.l}^{\left(t_{2}\right)},\cdots ,w_{.l}^{\left(t_{k}\right)}),$$ (2)

where $w_{.l}^{\left(t_{k}\right)}$ denotes the *l*th column of *W* ^(*t*_*k*_)^.

Each basis can be associated with a cluster of words; for each *l*th column at time *t*_*k*_, if

$$w_{il}^{\left(t_{k}\right)}>\delta ,$$ (3)

then we define that the *i*th word belongs to the *l*th cluster, where *δ* > 0 is the threshold. Note that this clustering method allows overlapped clustering, i.e., some of the elements can belong to several clusters. Thus, we obtain a sequence of the *l*th clusters $W_{l}^{\left(t_{1}\right)},W_{l}^{\left(t_{2}\right)},...,W_{l}^{\left(t_{k}\right)}$, where $W_{l}^{\left(t_{k}\right)}=\left\{\omega_{i}\in W|w_{il}^{\left(t_{k}\right)}>\delta \right\}$. This sequence can be interpreted as a temporal development of the set of words characterising the *l*th topic of collective attention.

### Experiment on Synthetic Data

As examples for solving the time sequential problem, firstly we conduct two numerical experiments using two types of synthetic data. The first experiment is to simply demonstrate whether our algorithm can classify matrices reasonably, and can track temporal development within each cluster. Moreover, we aim to compare our algorithm with a simple NMF. The second experiment is to test whether our algorithm can work, even if clusters merge or divide. The first data consist of randomly generated matrices, for simply simulating time sequential evolution. The second data are also randomly generated matrices, but these represent a merge or division of clusters.

**Fig 2:**
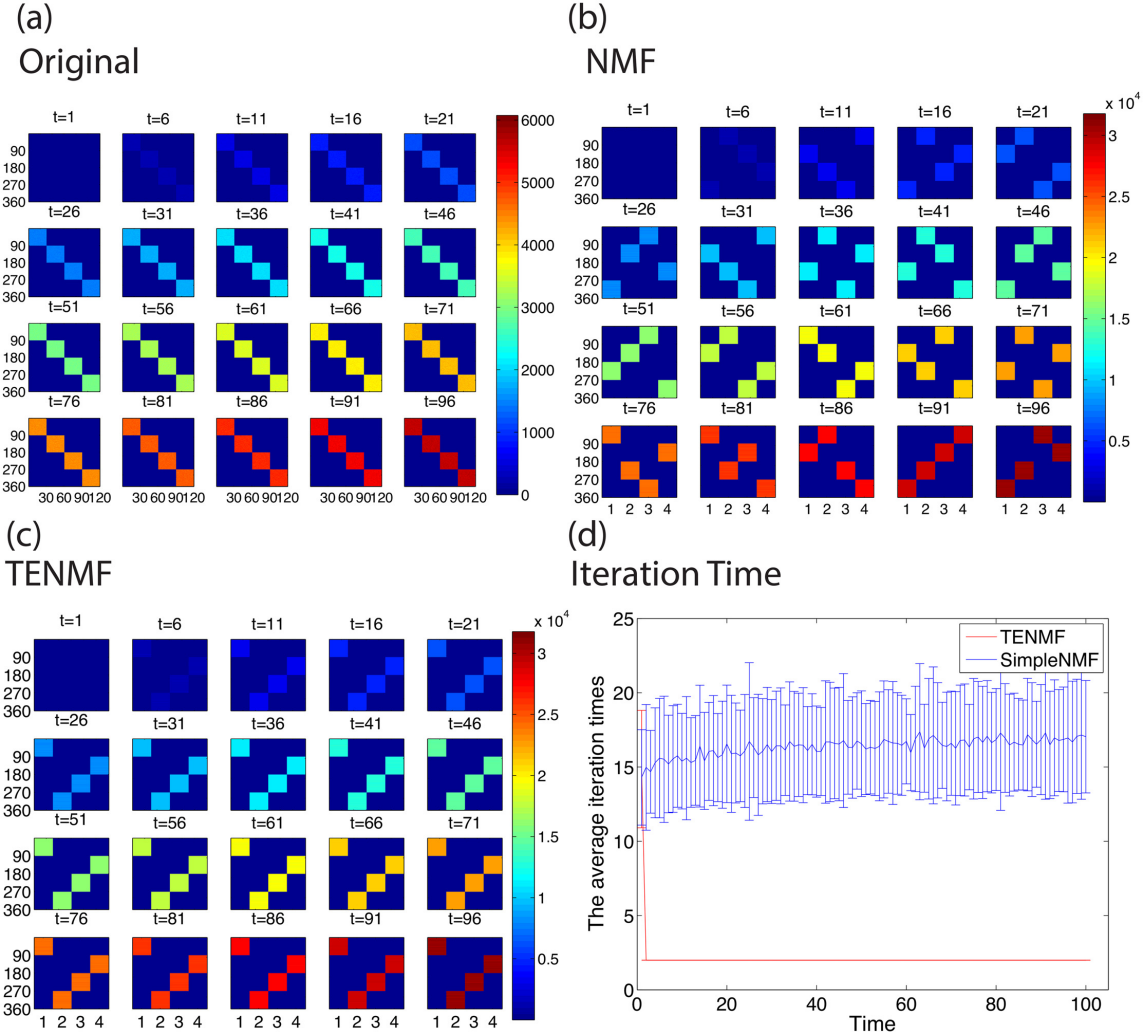
Comparison of Time Evolution Nonnegative Matrix Factorisation and simple Nonnegative Matrix Factorisation. (a) Snapshots of the original synthetic data for time-sequential matrices *V* ^(*t*_*k*_)^. Time evolves from left to right, and from top to bottom. The vertical and horizontal axes correspond to rows and columns of the matrices, and the values of the elements are represented by colour. We generate matrices such that the four equally sized blocks are filled with numbers that follow a Poisson distribution. (b), (c) Snapshots of the matrices *W* ^(*t*_*k*_)^ decomposed by (b) NMF and (c) TENMF with *r* = 4. Both NMF and TENMF decompose the original matrices properly, in the sense that each column of *W* ^(*t*_*k*_)^s corresponds to one block in the original matrices. On the other hand, each column of *W* ^(*t*_*k*_)^s decomposed by TENMF does not change the corresponding cluster. Moreover, the elements in one column evolve as the block evolves, which means each column in *W* ^(*t*_*k*_)^ can track the growth of the corresponding blocks in the original. (d) Iteration times required for decomposing *W* ^(*t*_*k*_)^ by NMF and TENMF. The number of iterations are counted for 200 runs, and the mean value and standard deviation are shown with error bars for each *k*. We can see that NMF requires more iteration time than TENMF. Moreover, NMF has larger variance than TENMF. This result shows that TENMF exploits the solution at time *t*_*k*_ as a good initial guess for the nearest locally optimal solution at time *t*_*k*+1_. This also means that TENMF respects the temporal similarity between the solutions at time *t*_*k*_ and *t*_*k*+1_.

The experimental result of the first setting is shown in Fig 2. Fig 2 (a)–(c) shows the original time-sequential matrices *V* ^(*t*_*k*_)^ and matrices *W* ^(*t*_*k*_)^ decomposed by the simple NMF and TENMF, respectively. The original matrices have four blocks in each matrix, and the values in the blocks gradually increase as time evolves (see Methods for the details).

We can observe that the TENMF algorithm can track the growth within each cluster properly, in comparison to NMF, which decomposes matrices arbitrarily. In Fig 2 (c), one column of *W* ^(*t*_*k*_)^s corresponds to one block in the original matrices consistently: For example, the first column whose 1st to 90th elements have values corresponds the upper left 90 × 30 sized block in the original matrices. In addition, each cluster can track the growth of the elements in the matrix. On the other hand, simple NMF loses the connection among time sequential matrices, i.e., in matrices decomposed by NMF we cannot observe that rows correspond to one cluster through elapsing times.

Fig 2 (d) plots required times for the convergence of simple NMF and TENMF. We can see that NMF requires more iteration time than TENMF. This result shows that if we employ the result of the previous time *t*_*k*_ as an initial condition, TENMF has less time to converge for the current time *t*_*k*+1_ in comparison to NMF. Moreover, NMF has larger variance than TENMF. The variance of time that results in NMF might be caused by the fact that initial values are randomly chosen, whereas TENMF converges in less time, and has almost no variance of convergence time. This experimental result supports our assumption that TENMF exploits the solution at time *t*_*k*_ as a good initial guess for the nearest locally optimal solution at time *t*_*k*+1_.

**Fig 3:**
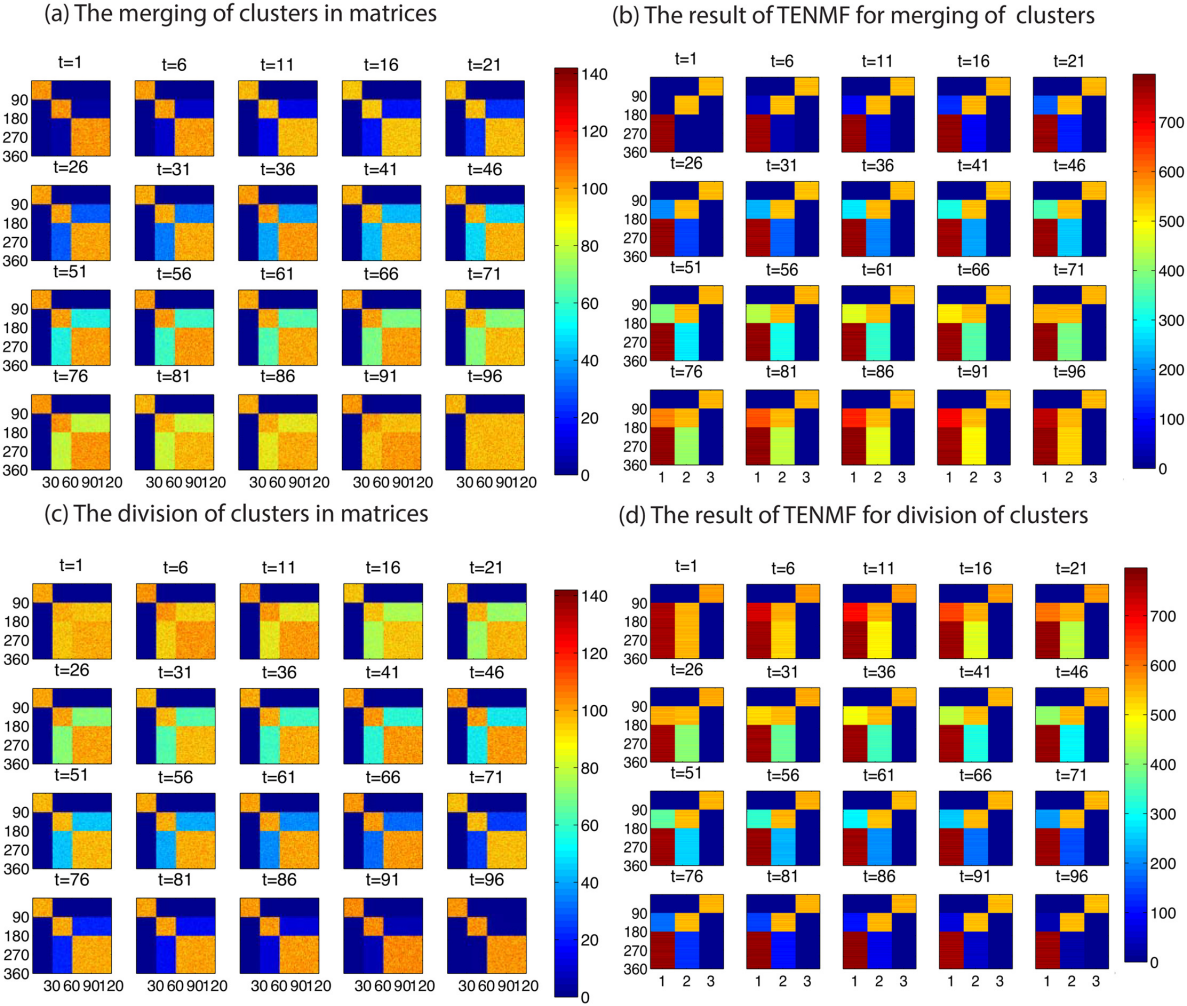
Simulation of TENMF for merging and division in a matrix. TENMF learns the merging and division of clusters. (a), (c) Snapshots of time-sequential matrices *V* ^(*t*_*k*_)^s describing (a) merging and (c) division of clusters. (a) There are three clusters at time *t* = 1, which are represented by three blocks aligned diagonally in the matrix. As time evolves, two of the three clusters gradually merge with each other, and finally constitute a single cluster at time *t* = 100. (c) The reversed sequence of the matrices in (a). (b), (d) Snapshots of *W* ^(*t*_*k*_)^s decomposed by TENMF from the time-sequential matrices shown in (a) and (c), respectively. (d) Our algorithm tracks the division of the original matrices, since the 2nd column, which is filled with relatively low values, assume the role of tracking the growth of the elements in the cluster, whereas the corresponding part of the original cluster, a part of 1st column, disappears gradually. In addition, our algorithm can track the merging of the original.

Fig 3 shows the result of the experiment in the second setting. In order to simulate merging, we firstly generate three blocks, and we gradually increase random values in two other blocks so that there seem to exist two blocks at the final state. For simulating division, we perform the same procedure backwards.

We can see that TENMF can track the changes of clusters, even if the cluster has been merged or divided. Fig 3 shows that if clusters are divided in the original matrices, then the 2nd column, which is filled with very low values, assumes a role of tracking the growth of the elements in the cluster, whereas the corresponding part of the original cluster, a part of 1st column, disappears gradually. If clusters merge, then two of the original clusters grow, but one with high values, and the other with low values. We suppose that the cluster with high values mainly represents the merges of the two groups, and the cluster with low values remains due to the effect of using the previous clustering results as the initial conditions.

### Application to Real Data from Twitter

In order to demonstrate our algorithm using real data, we used data collected from Twitter. The goal of this experiment with Twitter data is to classify users properly, and to track the growth of the elements within each cluster reasonably. We analyse 11,418,600 tweets, posted from 4th March 2011 to 16th March by 438,464 users, and 2,319,874 tweets posted from 1st June 2010 to 17th June 2010 by 438,464 users. These users tweeted mostly in Japanese. From the datasets, we picked up approximately 2,000 words, and created one matrix for each day. From these matrices we extracted word clusters using the TENMF algorithm with *r* = 4. The score of the *i*th word in the *l*th cluster at time *t*_*k*_ is defined as $w_{il}^{\left(t_{k}\right)}$. See Methods for the details of the experimental settings and the datasets.

**Fig 4:**
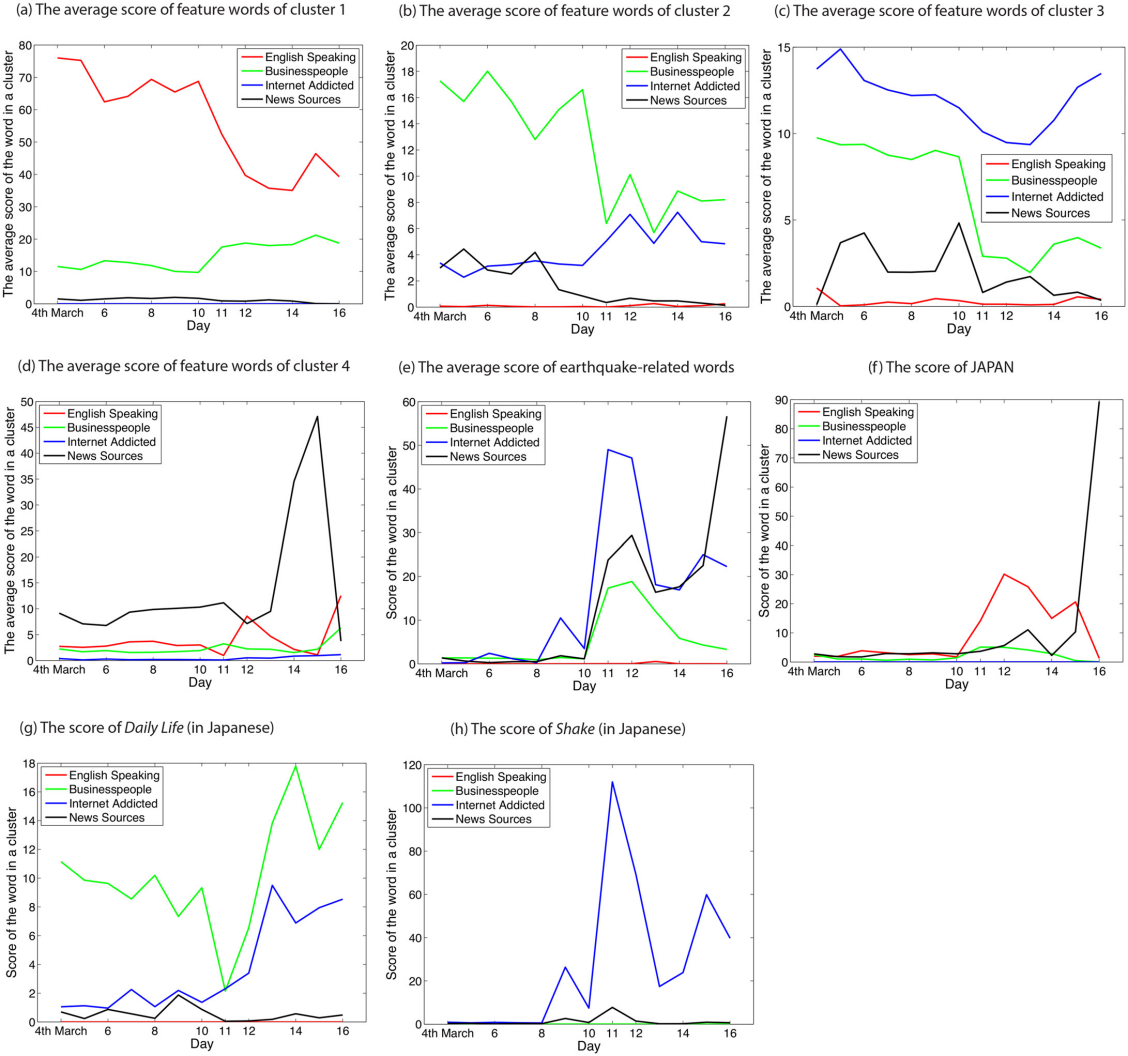
Scores of feature words and earthquake-related words for each cluster before and after Japanese earthquake. We analyse tweets that are collected in the interval of one week before and after 11th March 2011, when huge earthquakes occurred in Japan. (a)–(d) Average scores of the feature words shown in Fig 1. All words shown as feature words in Fig 1 represent characteristics of each cluster well, since all the average scores of the feature words are greater than the others. (e)–(h) Scores of some earthquake-related words within each clusters introduced in Fig 1. (e) Everyone mentions earthquake-related words, possibly because the earthquake was too big for any group of people to ignore. (f), (g), and (h) Some words have a spike mainly in affiliated clusters. For instance, the word *shake* has a spike only in ‘English speaking’. Details are discussed in the main text.

First, we illustrate the results from 4th to 16th March 2011 in Table 1 and Fig 4. Italic words here signal that they are originally in Japanese, and translated into English by the authors. We interpret the kind of people that are represented by clusters, by picking up words manually from those that have a higher value in the element of *W* ^(*t*_*k*_)^ in the cluster than the others. Fig 4 (a)–(d) shows that these manually picked-up representative examples of words have higher scores in each cluster than the others.

**Table 1:** Representative example of words belonging to clusters obtained by TENMF before and after Japanese earthquake.

	Representative Words between 4th and 16th March 2011
Cluster 1	No, we, by, YouTube, ⌃⌃, *lol* (笑)
Cluster 2	*regime* (政権), *a Diet member* (議員), *tired* (疲れ), *business routine* (業務) *society* (社会), *regulation* (規制), *work* (仕事)
Cluster 3	*madoka* (まどか) (a cartoon character), wwwww (lol, but more Internet-slangy) nico (popular video-sharing service in Japan)
Cluster 4	*asahi* (朝日), *yomiuri* (読売), *mainichi* (毎日), (These three are news media in Japan) *newspaper* (新聞), *article* (記事), *news* (ニュース)

To conduct the experiment, we collect tweets from 4th to 16th March 2011: the period for the huge earthquakes in Japan. Clusters represent some characteristics of user groups. We list the words with high scores that might represent the characteristics of each cluster. Italic words here are originally in Japanese shown in parentheses, and translated into English by the authors. If further parentheses are attached, the words are explained. Cluster 1 can be interpreted as ‘English speaking’ users or ‘symbols’ everyone uses. Cluster 2 can represent ‘businesspeople’. Cluster 3 can represent ‘Internet addicted’ users. Cluster 4 can represent ‘news sources’.

From Table 1, Cluster 1 can be interpreted as ‘English speaking’ users or ‘symbols’ everyone uses, since most of the words are written in English, or are symbolic words such as *lol*. Cluster 2 can be interpreted as ‘businesspeople’, since most of the words are related to social topics such as *regime* or a *Diet member*. The words belonging to cluster 3 can be tweeted by ‘Internet addicted’ users, since most of the words are related to Internet slang, or an Internet familiar web service. Cluster 4 can be interpreted as ‘news sources’, since the words are the names of news media, or are related to news.

Some scores of picked-up words appear in Fig 4 (e)–(h). We plot the temporal development of the score of the selected *i*th word in each cluster, i.e., we plot the *i*th element of Eq (2). Fig 4 (e) shows the average scores of *safe*, *refuge*, *nuclear plant*, *earthquake*, and *getting suffered*. We can observe that every cluster mentions earthquake-related words after 11th March. A small spike can be seen on 9th March, when a big earthquake also occurred as a foreshock for 11th March. Fig 4 (f) shows scores of JAPAN, Fig 4 (g) shows *daily life*, and Fig 4 (h) *shake*. From the results, we can recognise that each of the words has a higher score in a cluster that can be related to the word. In Fig 4 (f), the word JAPAN has a higher score in English speaking users. ‘English speaking’ users might care about such a huge earthquake, and mention JAPAN. In Fig 4 (g), the word *daily life* has a high score after 12th March. Before the earthquake, *daily life* in ‘businesspeople’ has a higher score than others. It then drops on 11th March, and from 12th March gains more than before 10th March. This might be because ‘businesspeople’ lived according to their daily lives before 10th March, then did not mention *daily lives* due to being affected by the huge earthquake on 11th March, and in the days following 12th March they lived in the aftermath of the earthquake, and began to wonder when they would get back to their daily lives. In Fig 4 (h), *shake* displayed a higher score in ‘Internet addicted’ users than the others. This high score reflects a custom among Twitter users in Japan; that is, if users experience an earthquake and use Twitter, then the users tend to tweet ‘has got shakes’ [20].

**Fig 5:**
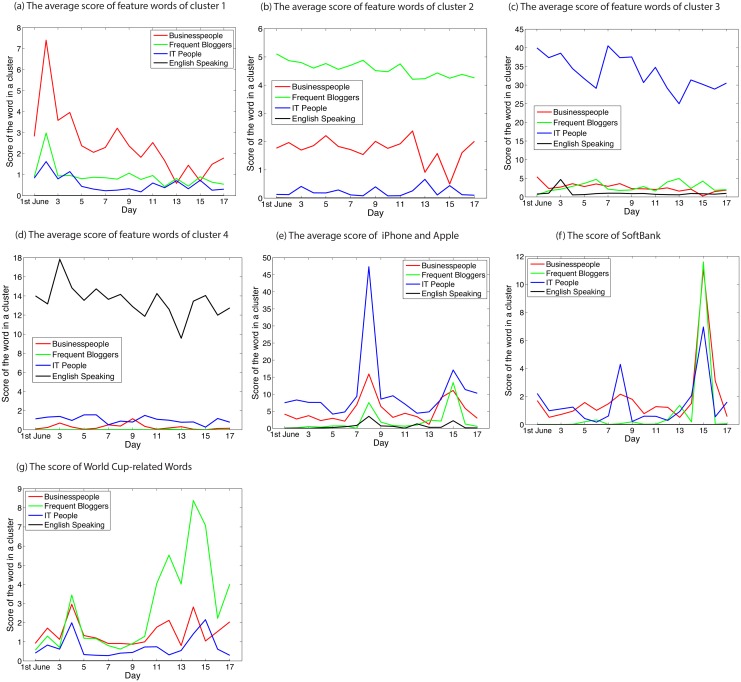
Scores of feature words and iPhone-related words for each cluster before and after iPhone announcement. We analyse tweets that are collected in the interval of almost one week before and after 7th June 2010, when apple announced new iPhone. (a)–(d) Average scores of the feature words shown in Fig 1. All words shown as feature words in Fig 1 represent the characteristics of each cluster well, since all of the average scores of the feature words are greater than for the others. (e)–(g) Scores of some picked-up words. (e) Everyone mentions iPhone-related words, possibly because the iPhone was also a big event for everyone. (f) The score of the name of the only provider of the iPhone at that time shows a spike on 15th June, the first day that the iPhone could be ordered. (g) FIFA World Cup-related words. This method also tracks the event of the World Cup held in the same period as iPhone announcement. Details are discussed in the main text.

Second, we show the results from 1st to 17th June 2010 in Table 2 and Fig 5, drawn in the same manner as Table 1 and Fig 4, respectively. The representative words for each cluster are shown in Table 2. Cluster 1 can represent ‘businesspeople’ for the same reason as in the case of the earthquake, because the representative words for cluster 1 are similar to those for cluster 2 in the huge earthquake. Cluster 2 can represent ‘frequent bloggers’, i.e., a cluster describing things related to daily life, since the words are related to daily activities, such as *going home* and *meal*. Cluster 3 can represent ‘IT people’, since the words are related to the names of web services or IT products. Cluster 4 can represent ‘English speaking’ users, since most of the words are in English. The reason why the representative words represent basic vocabularies might be that most of users in our dataset tweeted in Japanese, and it is only possible to detect basic vocabularies as a collective attention. The scores of these words are depicted in Fig 5 (a)–(d), which shows that these words have higher scores in each cluster than the others.

**Table 2:** Representative example of words belonging to clusters obtained by TENMF before and after iPhone 4 announcement.

	Representative Words between 1st and 17th June 2010
Cluster 1	*regime* (政権), *minister* (大臣), *Prime minister* (首相), *society* (社会) *employee* (社員), *sales* (営業), *industry* (産業)
Cluster 2	*lunch* (ランチ), *hungry* (空腹), *going home* (帰宅), *part-time job* (バイト), *room* (部屋), *meal* (飯), *train* (電車), *car* (車)
Cluster 3	IT, web, Google, iPad, Amazon, YouTube, *movie* (動画)
Cluster 4	what, good, like, show, love, have, go

To conduct this experiment, we collect tweets posted from 1st to 17th June 2010, when apple announced new iPhone. The notation in this Table is the same as Tabel 1. Cluster 1 can be interpreted as ‘businesspeople’ or people interested in ‘politics’. Cluster 2 can represent ‘frequent bloggers’. Cluster 3 can represent ‘IT people’. Cluster 4 can represent ‘English speaking’ users.

Fig 5 (e)–(g) shows the scores of picked-up words. Fig 5 (e) shows the score of iPhone and Apple. We can see that every cluster mentions iPhone-related words in the period of 8th and 15th June, when two iPhone-related events occurred. This result implies that this iPhone announcement event is also a big event for everyone. The iPhone-related words have a large spike on 8th June. This is probably because people mentioned the new iPhone, which was announced in 7th June. This apparent delay might be due to the fact that users from the data we use in the experiment are mainly Japanese, and Apple held a presentation announcing to release iPhone 4 on 7th June USA time, while the presentation occurred on 8th June in Japanese time. In addition, the words iPhone and Apple also show a small spike on 15th June. This small spike might be a consequence of the fact that people could order their new iPhone from this date in Japan. Fig 5 (f) shows the score of the word SoftBank, and we can see a spike on 15th June. SoftBank is a Japanese telecommunication company, and the only one provider of the iPhone in Japan at that time. This spike might be due to tweets mentioning the ordering of iPhones. Furthermore, we can recognise that it has a higher score in ‘businesspeople’ and ‘frequent bloggers’ than in ‘IT people’, whereas the iPhone-related words have a higher score in ‘IT people’. This result implies that ‘IT people’ cared about the iPhone itself more than ‘businesspeople’ and ‘frequent bloggers’, while those groups cared more about ordering the iPhone than ‘IT people’. Fig 5 (g) plots the average score of FIFA World Cup-related words: *world cup*, *soccer*, *Japanese national team*, *coach*, *goal*. This average score has a spike in ‘businesspeople’ and ‘frequent bloggers’ on 14th June, and in ‘IT people’ on 15th June. The reason why World Cup-related words do not have any high scores in ‘English speaking’ is probably because the selected words are all in Japanese. The spike on 14th June might be because the Japan vs Cameroon match was held from 23:00 to 1:00 of that day. The spike on the 15th in ‘IT people’ implies that they dedicate less attention in real-time, and rather checked after the match ended. Furthermore, we can say that Fig 5 (g) illustrates that our proposed algorithm can also track other events, besides those that we focused on during this period.

## Discussion

In this setting for matrices, in which the rows represent words and the columns represent users, we classify words into semantic groups by using Nonnegative Matrix Factorisation to classify users. This method is more reasonable than clustering users, for the following reasons. First, we have little clue towards understanding what characterise for the group of users if we classify them, unless we access each user belonging to the cluster and analyse each user carefully. It would be easier and more valuable if we classify the words into a semantic group and understand the characteristics of the cluster by the attributed words. Second, from the perspective of collective attention, the values of scores have little meaning if we classify the users. A value of scores in *W* ^(*t*_*k*_)^ relatively follows the frequency of the occurrence of words in tweets within distinct groups if we classify the words. On the contrary, if we classify users, then a value in *W* ^(*t*_*k*_)^ would indicate some sort of measure for the contribution of a particular user to the cluster. We have little interest in this contribution value of users, in terms of collective attention on Twitter.

In our demonstration using synthetic data and real data from Twitter, we set ranks manually. From the clustering problem perspective, the rank of the matrix represents the number of clusters. In general, clustering problems such as the K-means algorithm, EM algorithm, and Self-Organising Map have an issue that the number of clusters should be set manually in advance, as in the case of NMF. For NMF, the rank decision problem has been studied [21–24]. Our framework, however, requires prior knowledge about the data in advance in order to set the rank. In a synthetic data experiment, we know which rank is suitable in advance; but in our experiments using the Twitter data, we tune this rank parameter to the value which we can obtain the most interpretable results. This experimental setting for Twitter data means that we require prior knowledge about Twitter and Japanese users in order to apply the proposed method. As a future direction, it would be valuable if we formulate mathematical frameworks to set a rank in this problem. Such frameworks would help to analyse the data requiring lesser prior knowledge.

In order to make a matrix representing Twitter data, we need words in advance. The words should include both the word mentioning topics of collective attention, and the usual words characterising the clusters. In our experiments, we take the around 2,000 of the most frequent words from the perspective of occurrences in tweets. Subsequently, in the results, we manually pick up some words that might represent the characteristics of the clusters, which could add bias to our interpretation. One direction to reduce this bias would be to let crowds label each cluster using a crowdsourcing platform such as Amazon Mechanical Turk. Another direction is to automate this process of interpretation by a natural language processing technique, which, however, would be a future direction.

It is worth to mention the comparison with other well-known document clustering algorithms: Probabilistic Latent Semantic Indexing (PLSI) [25, 26] and its generalised algorithm, Latent Dirichlet Allocation (LDA) [27]. PLSI and LDA are said to be topic models, where each document can be seen as a mixture of various topics. PLSI yields a theoretically equivalent result to NMF [28]. LDA further assumes to have a Dirichlet prior, and LDA is a more complicated model than PLSI, and therefore than NMF, in terms of number of parameters to tune. However, in order to keep analysis simple, we use NMF in this study and extend to a time-sequential model.

Our method can be applied to other data such as social media analytics, finance, and seismology, as long as the data can be described as time-sequential matrices. In this work we focused on tracking the time-evolution of collective attention clusters in social networking sites. However, we can apply our methods to the problem of how the targeted elements evolve within the affiliated cluster. For instance, if we apply our method to earthquake data, we may know some clusters of earthquakes, which can be considered to happen for the same kind of causes, e.g., active faults.

Moreover, our method can be used to detect origins. As discussed before we require prior knowledge in order to tune a rank. The result is shown in Fig 3, however, that when time is evolved backwards, TENMF outputs the almost same result as the original one. This result indicates that if the number of clusters is known in advance, then we can identify the origin of merging and division. This could be applied for biological development and evolution for the identification of the original groups of the current cluster of groups; e.g., the development and evolution of birdsong, and the ontogeny of speech and language [29–31].

On several points, our work is distinguished from the previous related works. So far, since the Internet traces large amount of human behaviour, several aspects of user behaviour in websites have been studied in computational social science [1, 32]. Google search histories are used to predict epidemics such as flu and dengue fever, [33–36] and to analyse the stock market or bitcoin trends [37, 38]. Facebook data are used to analyse desicion making on voting [8] and information diffusion [39], and Wikipedia sources are used to predict the sales of movie tickets [40] and a stock market [41]. In particular, due to the real time nature, Twitter have drawn the attention and many researchers have studied human behaviour using its data from different standpoints, such as the analysis of general outlooks from Twitter [3, 4], information diffusion [5, 42–44], the detection of emerging topics [45, 46], the credibility of information [6, 47], the detection and tracking of real-world events [20, 48, 49], interaction with social media and real-life events [7, 50], collective attention [9, 10, 51], the estimation of demographics of users from web contents [52–54], and language analysis [55, 56]. Previous research, however, has been focused on one of the following characteristics: what is an emerging collective attention, who is talking about it, or how it evolves or shrinks. On the other hand, our work proposes an algorithm to characterise these three points at the same time.

Another well developed research area related to present work is matrix decomposition. Principal Component Analysis [57], Vector Quantisation [58, 59], and NMF [12] are classical methods of matrix decomposition. The advantage of NMF is nonnegative constraints, which yield an intuitive interpretation unlike other matrix decomposition methods. Thus, NMF has been studied from many perspectives [15, 60]. The foundation of NMF has been researched, such as the algorithm [16, 61–63], the rank decision problem, including sparseness constraints [21–24], and the initialising problem [17–19]. NMF has been applied to clustering problems [64, 65], such as document [66, 67], music analysis [68, 69], and the community detection problem [70, 71]. Online techniques for NMF [72, 73] are related to our work in terms of dealing with time-sequential data. However, they have previously dealt with the efficiency of computation if one matrix is extended by new signals, whereas we aim to track the temporal development of time-sequential matrices.

In conclusion, we have proposed a new method for tracking the temporal development of collective attention within each distinct user group in Twitter, by *Time Evolving Nonnegative Matrix Factorisation*, an extension of Nonnegative Matrix Factorisation. We have classified users by words in the tweets, and we tracked time-evolution of the tweet frequency of the words within each cluster. In our proposed method, we have described users and words in tweets posted within some interval by a matrix, and have used several matrices as time-sequential data. We have applied our Time Evolving Nonnegative Matrix Factorisation to these time-sequential matrices. We have also shown that our proposed algorithm performed adequately well for numerically generated matrices. Moreover, we were able to get some reasonable results and insights, using real data from Twitter.

There are several possible future directions. First although we have shown that our algorithm performed well on synthetic data, we do not have a criterion to evaluate our algorithm. Second although in this work we focused on tracking the time-evolution of collective attention clusters in social networking sites, we may apply our algorithm to other data such as social media analytics, evolutionary biology, seismology, and finance. Third our results should be affected by initial conditions since the objective function is multimodal. It would be good to know how initial conditions influence the result both theoretically and empirically. Finally it would be highly valuable to further explore the mathematical formulation of Time Evolving Nonnegative Matrix Factorisation. It is worth attempting to assign new regulation terms to the existing objective function [22–24], which is a widely used technique in the machine learning field [74]. It would be interesting to try a new objective function, reflecting the assumption that time-sequentially generated matrices change slightly.

## Methods

### Optimisation Formulation on Nonnegative Matrix Factorisation

In this section, we explain the NMF algorithm introduced in Ref. [16]. To find *W* and *H* in Eq (1), satisfying nonnegative constraints, we minimise the distance between *V* and *WH*, as follows:

$${\min\;∥V-WH∥}^{2}$$ (4)

s.t.W,H≥0, (5)

where we employ a simple element-wise Euclidean distance. In order to obtaion a solution, we optimise *W* and *H* alternately, as

$$H_{lj}\leftarrow H_{lj}\frac{{\left(W^{⊤}V\right)}_{lj}}{{\left(W^{⊤}WH\right)}_{lj}},$$ (6)

$$W_{il}\;\;\leftarrow \;W_{il}\frac{{\left(VH^{⊤}\right)}_{il}}{{\left(WHH^{⊤}\right)}_{il}},$$ (7)

until changes are sufficiently small. The monotonic convergence of the objective function is proven for the case in which we update as Eqs (6) and (7) [16]. Note that this algorithm converges to one of the local optimal solutions, since the cost functions are not convex for both *W* and *H*, although they are convex for each of them. We also remark that the solution is often not unique. If we find a solution *W* and *H*, another solution would be given by *WA* and *A* ^−1^ *H* as long as this satisfies the nonnegative constraints. In order to reduce arbitrariness, we adjust the *l*th column of *W* and the *l*th row of *H* to be such that the absolute norm of the *l*th row of *H* is 1, so that we can track how each cluster evolves.

### Synthetic Data

For each of the Figs 2 and 3, we generate 100 synthetic matrices *V* ^(*t*_*k*_)^ of size 360×120, for time *t* = 1,…,100. For Fig 2, we randomly generate values in the four equally-sized blocks from the Poisson distribution

$$P\left(X=k\right)=\frac{\lambda^{k}e^{-\lambda }}{k!},$$ (8)

with *λ* = 100*t*. For Fig 3, we generate three blocks that filled randomly by Eq (8) with *λ* = 100. We fill the two other off-diagonal blocks with the elements in which develop time-sequentially by setting *λ* = 100×*t*/100 in order to achieve a final state in which there exist two blocks in the matrix for the final time *t* = 100.

### Real Data from Twitter

To demonstrate our algorithm to real data, we used the publicly available data collected from Twitter via Twitter API. We use the same dataset as one used in Ref. [10]. This dataset is a collection of publicly available tweets in the period from 4th March 2011 to 16th March and from 1st June 2010 to 17th June 2010 in Twitter. Tweets are obtained from the collected users. The sampling of users of this dataset is conducted by snowball sampling; starting with 10 initial users who have large numbers of followers such as celebrities, we iteratively collect new users who get retweeted or replied by the collected users. Refer to Ref. [10] for more detail. We use 11,418,600 tweets posted in the interval of from 4th March 2011 to 16th March and 2,319,874 tweets posted in the interval of from 1st June 2010 to 17th June 2010 by 438,464 users, which are mainly Japanese tweets, to know the dynamics in Twitter when Japan had huge earthquakes in 11th March 2011 and iPhone announcement in 7th June 2010. We adhered to Twitter’s Terms of Use and Terms of Service in this study.

We firstly did morphological analysis of these tweets and decompose into words by MeCab [75] and choose the 2,032 frequently used Japanese nouns for the earthquake and alphabetical symbols in terms of occurrence, and made 2,256 words for the iPhone announcement in the same manner. We made one matrix for one day, i.e., 13 2,032×438,464 matrices for earthquake, and 17 2,256×438,464 matrices, whose columns represent users, rows represent words, and each element represents the occurrence of a certain words in tweets by a certain user within one day. We used randomly generated *W* ^(0)^ and *H* ^(0)^ for initial values, and conducted TENMF algorithm. Through the TENFM, a rank *r* was set to 4. We employed a threshold *δ* = 2 to pick up words affiliating to a cluster so that 8% words were selected for representing one of clusters.
